# Ant Diversity and Stratification in an Amazonian Rainforest

**DOI:** 10.1002/ece3.72793

**Published:** 2025-12-17

**Authors:** Jacques H. C. Delabie, Alain Dejean, Maurice Leponce, John T. Longino, Jérôme Orivel, Axel Touchard, Arthur Compin

**Affiliations:** ^1^ Laboratório de Mirmecologia CEPLAC/UESC/UFSB Ilhéus Bahia Brazil; ^2^ Departamento de Ciências Agrárias e Ambientais Universidade Estadual Santa Cruz Ilhéus Bahia Brazil; ^3^ Toulouse INP, CNRS, IRD, CRBE, Université de Toulouse Toulouse France; ^4^ UMR EcoFoG, AgroParisTech, CIRAD, CNRS, INRAE Université des Antilles, Université de Guyane Kourou France; ^5^ Institut Royal des Sciences Naturelles de Belgique Operational Directorate Natural Environment Brussels Belgium; ^6^ Evolutionary Biology and Ecology Université Libre de Bruxelles Brussels Belgium; ^7^ Department of Biology University of Utah Salt Lake City Utah USA

**Keywords:** ants, colony size, feeding habits, functional groups, nesting mode, sampling methods

## Abstract

This study focuses on species occupying the three strata of an Amazonian rainforest: the ground and leaf litter, the understorey and the canopy. We employed only two sampling techniques: Winkler extraction for ground‐dwelling ants and direct observations for understorey and canopy species on large branches cut off by a climber. We identified 494 ant species from 10 subfamilies and 77 genera over ≈3.0 ha (Chao1 = 607 species; 95% CI: 566–670 species). Although we found fewer arboreal ants compared to approaches using insecticide fogging, this study confirms similarities between the ant diversity in Amazonian and Mesoamerican rainforests. The functional traits of these ants (i.e., diet, nest‐site preference, population size of the colony) allowed us to identify seven clusters. Cluster 1 is a “hodgepodge” grouping arboreal or ground‐dwelling species with different‐sized colonies (76 species). Cluster 2 primarily includes small colonies of ground‐nesting generalist feeders (142 species). Cluster 3 comprises all arboreal species from the understorey inhabiting myrmecophyte domatia or palm trees plus arboreal species with medium‐sized colonies (37 species). Cluster 4 includes all territorially dominant arboreal ants plus one ground‐dwelling species (21 species). All fungus‐growing species belong to Cluster 5, which also contains ground‐nesting generalist feeders and generalist predators (148 species). All doryline army ants are grouped in Cluster 6 along with one ponerine known for its nomadic behavior (15 species). Almost all specialized predators belong to Cluster 7 (55 species); however, Cluster 5 includes two ponerine species that prey exclusively on termites. Based on a nonmetric multidimensional scaling (NMDS), we confirmed that the position of these clusters corresponded fairly well to the three forest strata. Thus, analyzing functional traits enables the trophic position of most ants and their place in the vertical strata of Neotropical rainforests to be determined.

## Graphical abstract

We examined the three strata of an Amazonian rainforest (e.g., the ground and leaf litter, understory and canopy) using only two sampling techniques and analyzed their functional traits. We identified 494 ant species from 10 subfamilies and 77 genera over ≈3.0 ha, showing that they are species rich and abundant, particularly at ground level. The functional traits of these ant species based on their diet, nest‐site preference, size of the colony, and the stratum where they live allowed us to identify seven clusters.

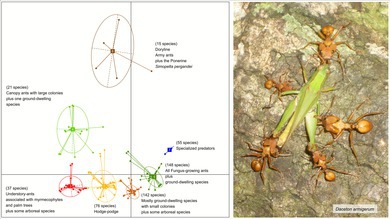

## 1 Introduction

Due to warm temperatures and heavy rainfall, tropical rainforests exhibit a disproportionately high biodiversity compared to their surface area (Gallery 2014; Pillay et al. 2022). This fosters a high diversity of plants involved in various kinds of relationships with animals, mostly arthropods, whose numerous taxa occupy different trophic levels in these forests. Indeed, temperature and humidity are relatively stable at ground level and to a lesser degree in the understorey if compared to the canopy which is particularly exposed to the sun and wind. Thus, large differences in insolation, temperature, and humidity occur in the strata which can be seasonal or day‐by‐day (De Frenne et al. 2021; Deng et al. 2022; Nakamura et al. 2017). Solar exposure and air temperature increase with vertical height while relative humidity decreases (e.g., for every 10 m, temperature increases by 0.13°C and relative humidity decreases by 1.4%; Xing et al. 2022). These abiotic variations in environmental characteristics have repercussions on the vegetation structure generating a vertical spatial distribution in species composition for vertebrates and invertebrates as many of them are adapted to a stratum; that is, the ground, the understorey, or the canopy (Basham et al. 2019; Basset et al. 2012, 2015).

Among arthropods, ants are one of the most ecologically important animal lineages due to their diversity, with more than 14,000 species described and a very large number of individuals, so that they represent ≈12 million tons of dry biomass on Earth, more than both wild birds and mammals combined (Borowiec et al. 2025; Schultheiss et al. 2022). They have a major role in the functioning of tropical rainforests due to their abundance, high species richness, seasonal permanence and, for certain species, ecological dominance (Basset et al. 2023; Rico‐Gray and Oliveira 2007).

We took advantage of the numerous studies conducted on Neotropical ants over the last 30 years that, by providing information on their biology and ecology, enable functional groups to be identified. Syntheses on the functional groups of Neotropical ants can be found in Brandão et al. (2012) and Koch et al. (2019); for predatory ants see Dejean et al. (2025). This allowed us to conduct a comprehensive survey in an Amazonian rainforest in the hopes that gathering ants from all strata would provide insights into their species richness, permitting a better understanding of the complexity of the functioning of this ecosystem. We selected traits related to their feeding habits, nesting modes, and, because it is an important parameter in rainforest functioning due to large variations, the number of individuals living in ant colonies (i.e., from a dozen workers to several hundred thousand individuals). Species with large colonies include leaf‐cutting ants that are major defoliators, most army ants that regulate arthropod communities in and on the ground, and territorially dominant arboreal ant species (TDAAs) whose territoriality and predation enable them to eliminate defoliators (Blüthgen and Stork 2007; Dejean et al. 2025; Rico‐Gray and Oliveira 2007). We did not use morphological traits because their relationships with vertical stratification are not clearly discernable for Neotropical ants (see Vieira et al. 2025).

Although many studies dealing with Neotropical rainforest ant diversity have focused on only a single stratum, two studies focused on both the ground and the canopy. Using seven and six sampling techniques, respectively, Longino and Colwell (2020) gathered 539 species in Costa Rica, while Ryder Wilkie et al. (2010) collected 489 ant species in Ecuador. One comprehensive survey was conducted in Panama to record ants in all three strata (i.e., the ground, the understorey, and the canopy); using 11 sampling techniques, Leponce et al. (2021) recorded 405 ant species. Note that insecticide fogging was used to gather arboreal ants in these cases.

Because it deals with an old pristine Neotropical rainforest (Charles‐Dominique et al. 1998), the present study permitted us to record information on ant ecology and evolution via their adaptation to the stratum where they live, at least for most of them. Indeed, many ants are adapted to living in specific conditions. Ground‐dwelling species are frequently predatory; certain of them are even specialized in the capture of a single prey type, and, specifically in the Neotropics, fungus‐growing species also abound (Dejean et al. 2025; Vieira et al. 2025). However, leaf litter can accumulate between the fronds of palm trees or under epiphytes, permitting certain ground‐dwelling species to live there (Dejean et al. 2025; Rico‐Gray and Oliveira 2007). Coevolution occurred between ants and myrmecophytes living mostly in the understorey and myrmecophytic epiphytes in the canopy, the ants obtaining shelter in hollow structures called domatia and sometimes food (e.g., extrafloral nectar, food bodies); in exchange, they provide their host with protection and nutrients (Orivel and Leroy 2011; Rico‐Gray and Oliveira 2007). Specialized canopy ants are TDAAs and the ant species with smaller colonies they tolerate on their territory (Majer et al. 1994). The latter can live in association with epiphytes, in hollow twigs or branches, or are able to build their nests (Bomfim et al. 2025; Dejean et al. 2019; Rico‐Gray and Oliveira 2007). Consequently, tropical rainforest ants are characterized by a vertical stratification (Longino and Colwell 2020; Ryder Wilkie et al. 2010; Leponce et al. 2021; Vieira et al. 2025).

In this study, using species rarefaction curves, we first analyze the entire ant assemblage as well as that corresponding specifically to each stratum. Second, we attempt to show differences and overlaps in ant species composition between the three strata using proportional Venn diagrams and a nonmetric multidimensional scaling. Third, we use ant functional traits to identify what ant species belong to each of the three rainforest strata.

## 2 Materials and Methods

### 2.1 Study Site and Ant Sampling Methods

The Nouragues Ecological Research Station (4°05′N–52°41′W; https://cnrs‐nouragues.fr) is located in French Guiana where the climate is tropical moist (mean annual rainfall: 3000 mm; mean temperatures: 20.3°C–33.5°C) (Grimaldi and Riéra 2001).

**FIGURE 1:**
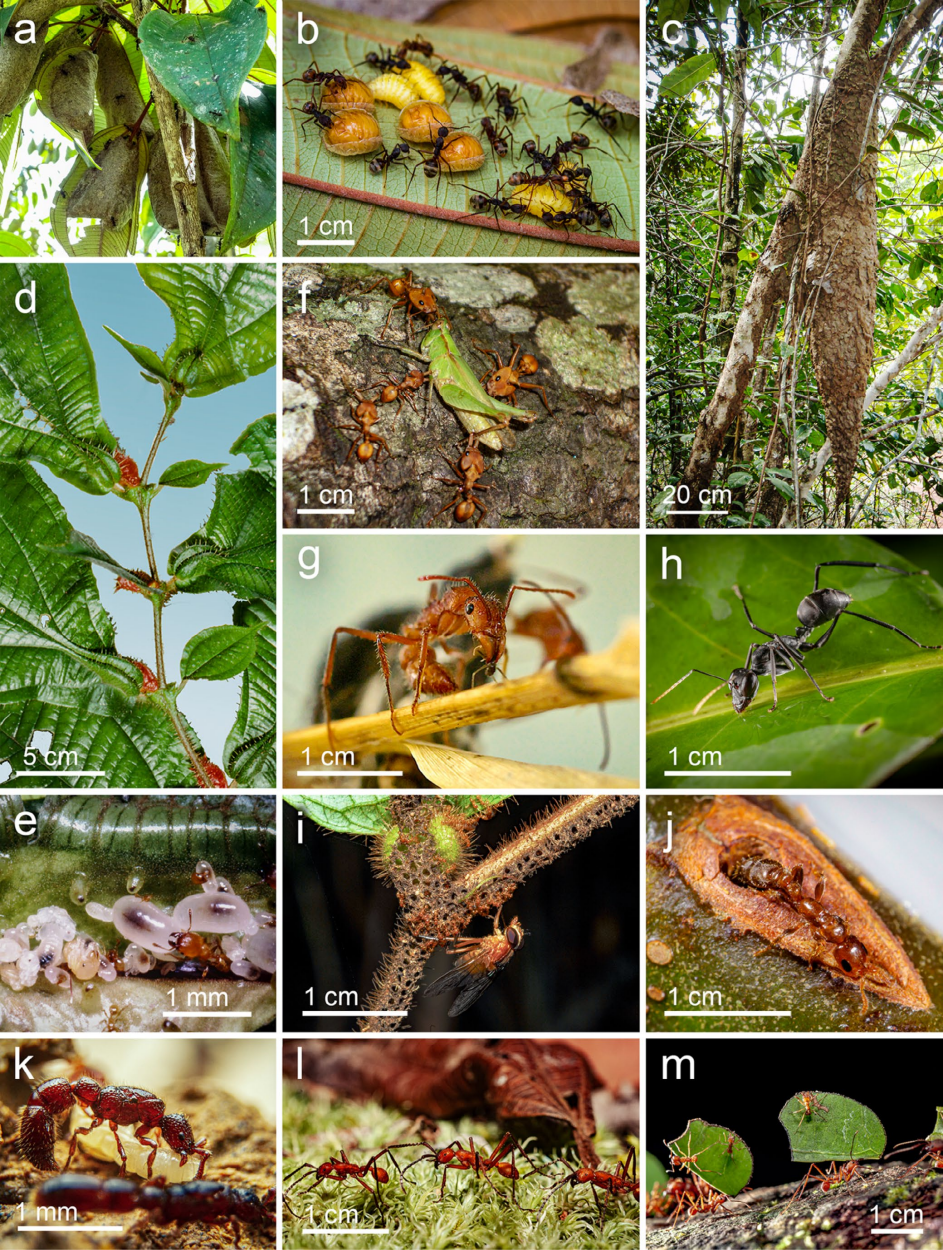
Neotropical ant species associated with different forest strata. (a) In the canopy, nests of *Dolichoderus bidens* under leaves and (b) workers tending ladybug larvae inside these nests; (c) nest of *Azteca chartifex* . (d) *Maieta guianensis*, a myrmecophyte from the understorey sheltering (e) *Pheidole minutula* in a leaf pouch. (f) *Daceton armigerum* workers preying on a locust in the canopy, (g) an *Ectatomma tuberculatum* capturing an *Azteca* worker and (h) a forager of *Gigantiops destructor* . In the understorey, (i) *Allomerus decemarticulatus* preying on a fly on a *Hirtella physophora*, (j) *Pseudomyrmex penetrator* leaving a domatium of *Tachigali paniculata*. Ground‐dwelling (k) *Neocerapachys splendens*, (l) *Eciton burchellii* and (m) *Atta cephalotes* workers transporting pieces of leaves. Photo credits: B. Corbara (a, b), A. Dejean (c–f, i), J.‐P. Lachaud (g), M. Leponce (h, l, m), A. Touchard (j) and E.A. dos Santos‐Neto (k).

Ground, understorey, and canopy studies were conducted over an area of approximately 3 ha during three field campaigns in 2011, 2014, and 2018 (Figure 1). We used the Winkler method recommended for invertebrate inventories in tropical rainforests where litter abounds (Delabie et al. 2021). We gathered 170 Winker samples of 1 m^2^ situated at a minimum interval of 10 m from nine transects covering 1.7 km. Each leaf‐litter sample was then placed in a cotton “litter sifter” (94 cm in length; 27 cm in diameter) to dry during 48 h. The ants climbed down the sifter in search of greater humidity and then fell into a tube containing 70° ethanol.

For canopy ants, we used the single rope technique (Steege 1998) to reach the crown of each tree, permitting us to cut off two to four large branches, while researchers remaining on the ground used entomological aspirators to collect samples of the ants crawling on the branches. To gather ants hidden in hollow twigs, we opened these twigs using pruning scissors. In addition, our study site included a treefall gap where a large dead trunk had fallen over, bringing down a live tree and lianas with it. We also systematically examined the bases of trees to look for nests of *Paraponera clavata* and *Ectatomma tuberculatum* (from the Paraponerinae and Ectatomminae subfamilies, respectively), as these ants forage arboreally. The whole study allowed us to monitor 169 trees whose identification is provided in Dejean et al. (2019).

Finally, understorey ants were collected by inspecting the debris accumulated between the fronds of 84 young palm trees, *Astrocaryum sciophilum*, that are relatively frequent in this area (Charles‐Dominique et al. 2003). We also looked for ants associated with eight understorey myrmecophytes. We examined 252 *Maieta guianensis* (Melastomataceae), 163 *Hirtella physophora* (Chrysobalanaceae), 75 *Cordia nodosa* (Boraginaceae), 61 *Cecropia obtusa* (Urticaceae), 38 *Tachigali paniculata* (Fabaceae), 30 *Tachia guianensis* (Gentianaceae), 18 *Tococa guianensis* (Melastomataceae), and 10 *Duroia longiflora* (Rubiaceae) (totaling 646 myrmecophytes). In each case, using pruning scissors, we completely opened two to four domatia to gather ant samples using an aspirator.

Ant samples were preserved in 70% ethanol for later identification in the laboratory. Voucher specimens were deposited in the collections of the *Laboratório de Mirmecologia*, CEPLAC/UESC/UFSB, Ilhéus, Bahia, Brazil; the Royal Belgian Institute of Natural Sciences, Brussels, Belgium; and the Department of Biology of the University of Utah, USA.

### 2.2 Ant Diversity and Distribution

Species rarefaction curves were plotted based on the species occurrences data matrices using EstimateS 9.1.0 software (Colwell 2016) with 100 randomizations of the sampling order without replacement. To estimate sampling completeness, the Chao1 nonparametric estimator of total species richness was calculated (Colwell et al. 2004).

To show differences and overlaps in ant species composition and richness between the three strata, we used proportional Venn diagrams, in which the area of each part of the rainforest (the ground, the understorey and the canopy) is proportional to the number of species it harbors (see also Table S1).

### 2.3 The Ant Functional Traits

We defined nominal categories for three functional traits based on studies of Neotropical rainforest ants as well as the results obtained here on their distribution throughout the three forest strata (Table S2; Compin et al. 2025). (1) Feeding habits (eight categories): granivorous; feed on extrafloral nectar or food bodies; honeydew feeders; pollinivorous; fungus‐growing; scavengers; generalist predators; specialist predators. (2) Nesting habits (seven categories): bivouac; ground nesting; leaf‐litter nesting; myrmecophyte or ant‐garden nesters; arboreal nesting in hollow twigs (nonmyrmecophytic trees); arboreal nesting in cavities; arboreal building carton. (3) Colony size: as in Leponce et al. (2021), the colony size of mature colonies is divided into five categories: < 300; 300–1000; 1000–10,000; 10,000–100,000; or > 100,000. Ranges for colony sizes were determined by two of the co‐authors (J.H.C.D. and J.T.L.) based on their own field studies and experience in taxonomy, in addition to bibliographic information (Bell‐Roberts et al. 2024; Burchill and Moreau 2016 whose online table S1 presents the ant colony size for 474 species).

The composition of the sampled community allowed us to build a [20 functional traits × 494 ant species] matrix. The scores corresponding to the functional traits ranged from “0”, indicating “no affinity” for a given trait category, to “3”, indicating “high affinity”.

We conducted a Fuzzy Correspondence Analysis (FCA) on this [ant species × traits] matrix. We then ran a *k*‐means clustering based on the Euclidean distance between the data points of the FCA and determined the number of clusters using the result obtained from the calculation of 23 clustering indices (Charrad et al. 2014) that corresponded to seven clusters (i.e., 8 times out of 23).

### 2.4 Distribution of Ant Assemblages and Functional Traits Along a Vertical Gradient

To determine the influence of the three rainforest strata on the ant species recorded, we conducted a nonmetric multidimensional scaling (NMDS) on a matrix based on the Jaccard dissimilarity index using 100 random starts due to the abundance of zeros in the data (McCune et al. 2002). The final stress value of 0.01 < 0.1 can be acknowledged as providing a good representation for a 2‐D configuration. We then ran a “Complete clustering” based on the Euclidean distance between the data points of the NMDS and determined three clusters using the result obtained from the calculation of 23 clustering indices (i.e., 14 times out of 23) (Charrad et al. 2014). These analyses were conducted using the Vegan and NbClust packages in R software (R Development Core Team 2024).

We then transposed the results of the Fuzzy Correspondence Analysis on that figure to show the positions of the different ant communities defined by the functional traits.

All analyses were carried out using the ADE4, NbClust, and ggplot2 packages in R software (R Development Core Team 2024).

## 3 Results

### 3.1 Ant Diversity and Distribution

**FIGURE 2:**
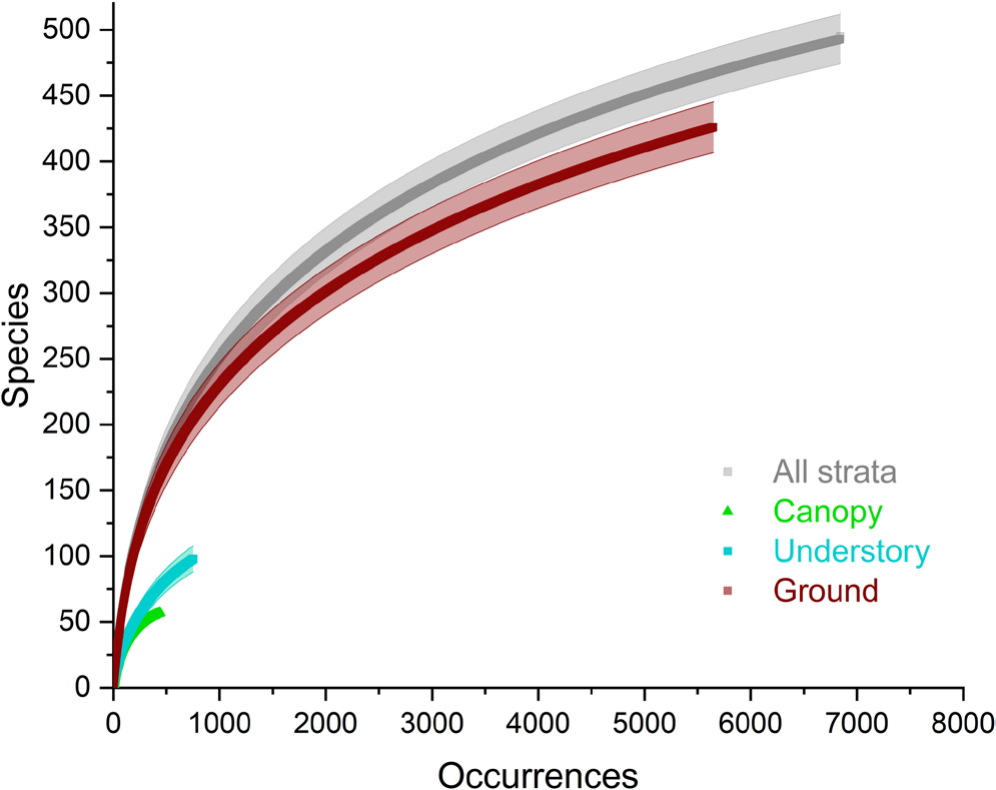
Sample‐based rarefaction curves for 494 ants (6843 occurrences for 1292 samples corresponding to 393 Winkler extractors, 169 trees, 84 palm trees, and 646 myrmecophytes; Table S1).

We recorded 494 ant species in total from the three strata and, according to the Chao1 estimator, we collected about 81% of the species present locally (Chao1: 607 species, 95% CI: 566–670). The rarefaction curves show the predominance of ground‐ and litter‐dwelling ants compared to those from the canopy, which are mostly represented by TDAAs that, due to their large colonies occupying several tree crowns, are comparatively less species‐rich. For understorey species, the shape of the curve is influenced by the litter‐dwelling species nesting in the debris accumulated between the fronds of the palm tree *Astrocaryum* while the other species are primarily “plant ants” associated with myrmecophytes (Figure 2).

**FIGURE 3:**
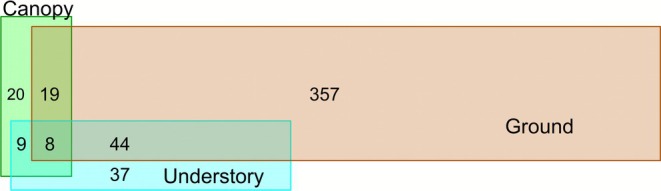
Venn diagram of the 494 ant species collected from the ground, understorey, and canopy showing differences and overlaps between the three strata.

The distribution of the ant assemblage between the ground, understorey and canopy shows that, of the 494 ant species gathered, 357 were ground‐ or litter‐dwelling species (Figure 3). In the understorey, the myrmecophytes in this stratum attract specific plant ants, whereas 37 litter‐dwelling species nest between the fronds of the palm tree *Astrocaryum*, as does the arboreal ponerine *Odontomachus hastatus* . We recorded 20 canopy specialist species, most of which are TDAAs. The other species were noted in two (44 and 9 species, respectively) or in all three strata (8 species) (Figure 3).

**FIGURE 4:**
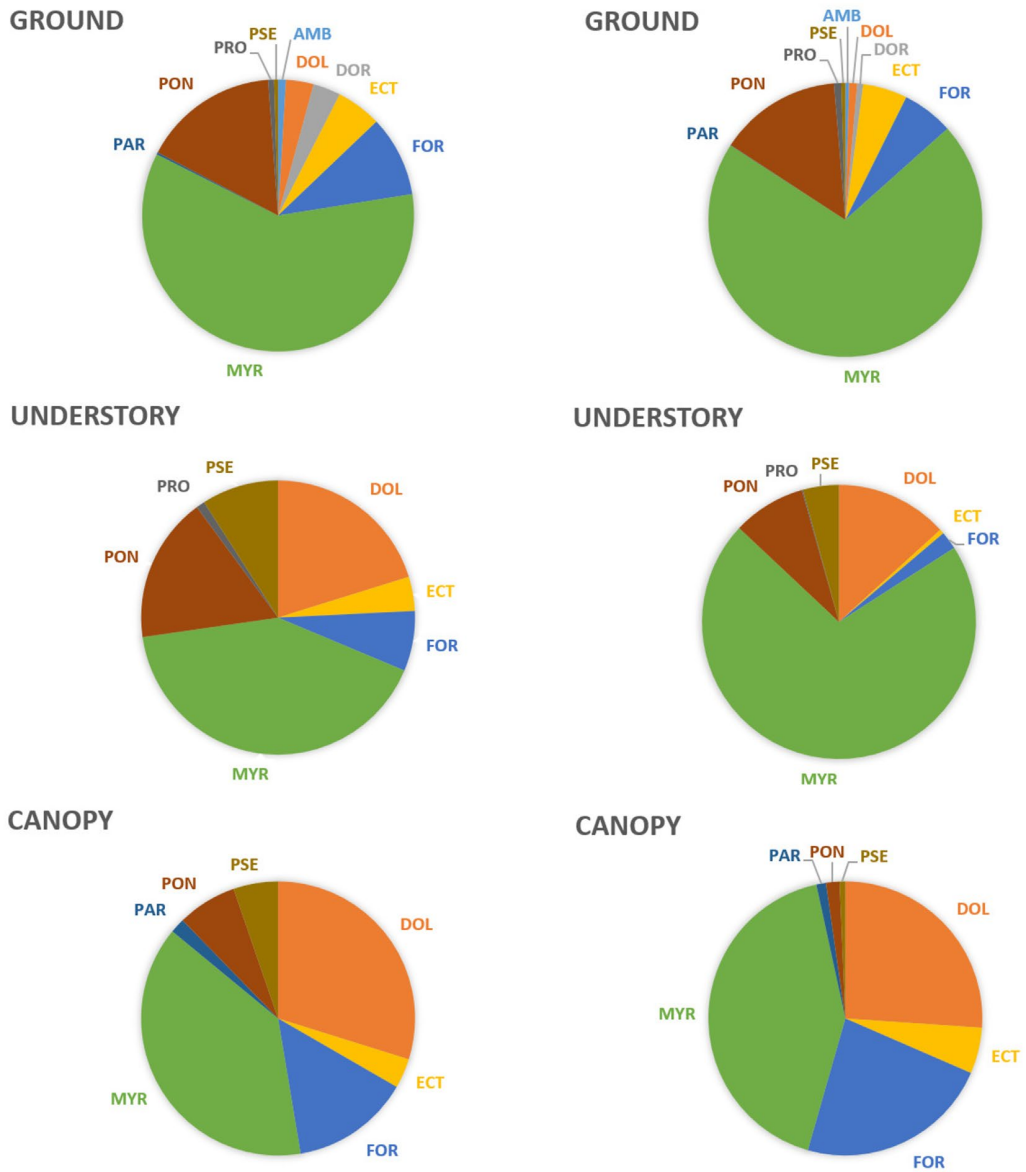
Number of ant species (left) and abundance (right) in the three strata of the Nouragues rainforest (Dol, Dolichoderinae; Ect, Ectatomminae; For, Formicinae; Myr, Myrmicinae; Par, Paraponerinae; Pon, Ponerinae; PRO, Proceratiinae; PSE, Pseudomyrmecinae).

The ground stratum was dominated by the Myrmicinae (primarily the genus *Pheidole*, with 93 species, followed by *Solenopsis*, with 24 species, for which identification is somewhat difficult) and the Ponerinae. We recorded a higher prevalence of Dolichoderinae in the understorey, where numerous myrmecophytic *Maieta guianensis* grow, most of them sheltering a colony of *Pheidole minutula* , so that this ant species, with 238 occurrences, was the most frequent in this study. Finally, the canopy, where the Myrmicinae are also dominant, hosts significant proportions of Dolichoderinae and Formicinae (Figure 4; Table S1).

### 3.2 The Ant Functional Traits

**FIGURE 5:**
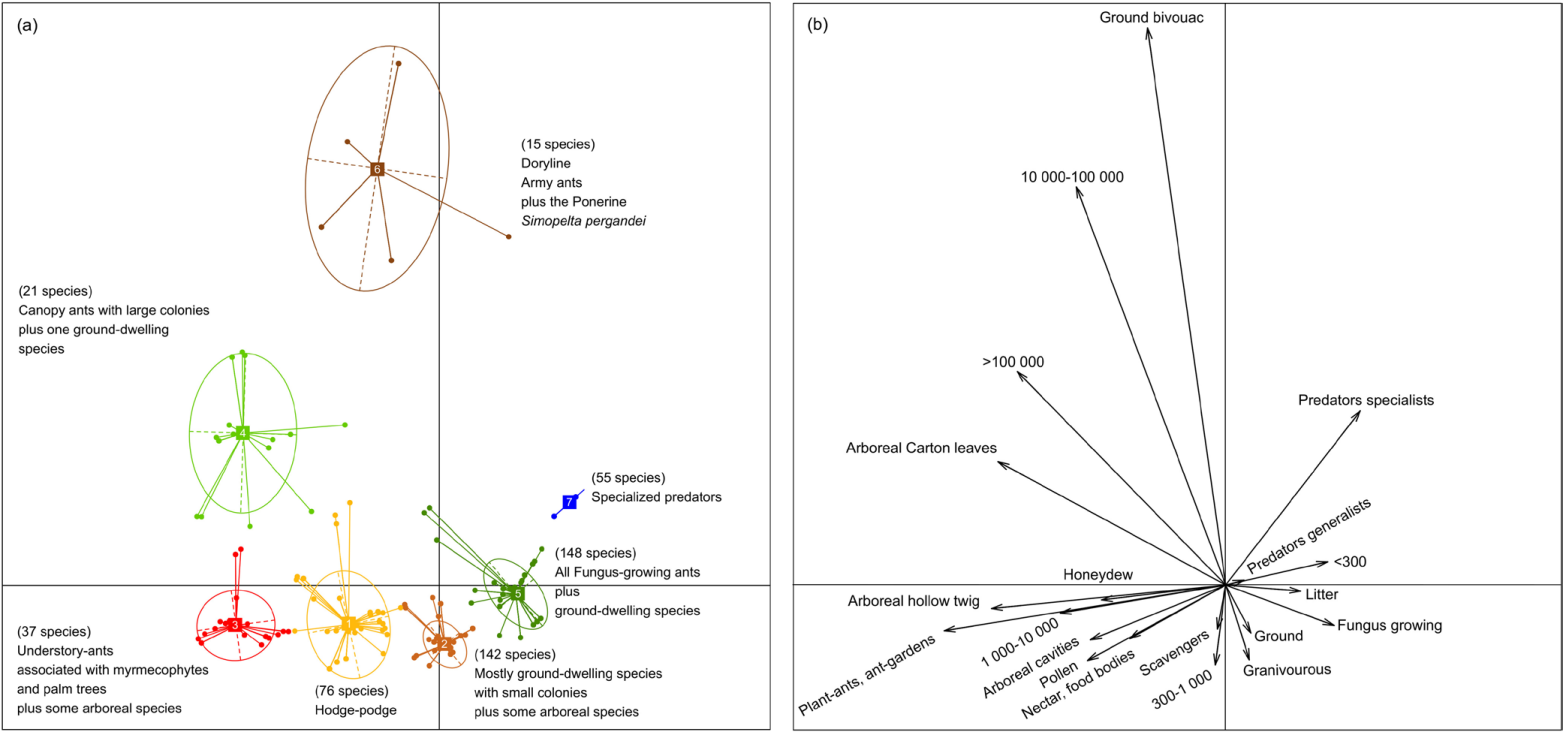
(a) Ordination of the functional traits composition of the 494 ant species recorded on the two first axes of the Fuzzy Correspondence Analysis showing seven clusters (6843 occurrences); the percentage of species in each cluster is noted between parentheses. (b) Ordination of the functional trait modalities on the two first axes of the FCA.

The functional traits of the 494 ant species recorded resulted in seven distinct clusters (Figure 5a,b; Table S2).

Cluster 1 encompasses a wide range of colony sizes (i.e., from < 300 to > 100,000 individuals) with generalist feeding habits, including arboreal and ground‐ or litter‐dwelling species (76 species in total).

Cluster 2 is primarily composed of small colonies (300–1000 individuals) that nest in the ground or litter, although seven ponerine ants from the genus *Neoponera* plus *Platythyrea sinuata* are arboreal. They are generalist feeders or exhibit both scavenging and generalist predatory behaviors. Most *Pheidole* species belong to this cluster (142 species in total).

Cluster 3 encompasses all 23 plant ants, or ants sheltering in myrmecophyte domatia, plus *Od. hastatus* that is associated with the palm tree *Astrocaryum*; all of them live in the understorey. Additionally, 14 other arboreal ants with colony sizes ranging from 1000 to 10,000 individuals are included in this cluster (37 species in total).

Cluster 4 comprises the 20 ant species which form large canopy colonies, primarily TDAAs (most containing > 100,000 individuals), but one ground‐nesting species with large colonies, namely *Wasmannia rochai* , also belongs to this cluster (21 species in total).

Cluster 5 consists of all 39 fungus‐growing ants, which are grouped with ground‐dwelling generalist‐feeding species or generalist predators that have small colonies (most with < 300 individuals) (totaling 148 species).

Cluster 6 encompasses all the doryline army ants recorded in this study, regardless of whether they are generalist or specialist predators, as they nest in bivouacs and have rather large colonies (1000–10,000; 10,000–100,000 or > 100,000 individuals). In addition, the ponerine *Simopelta pergandei* also belongs to this cluster due to its army ant‐like behavior (15 species in total).

Cluster 7 comprises almost all specialist predator species that nest in the ground or the leaf litter; all of them have small colonies (< 300 individuals) (55 species in total).

### 3.3 Distribution of Ant Assemblages and Functional Traits in the Three Forest Strata

**FIGURE 6:**
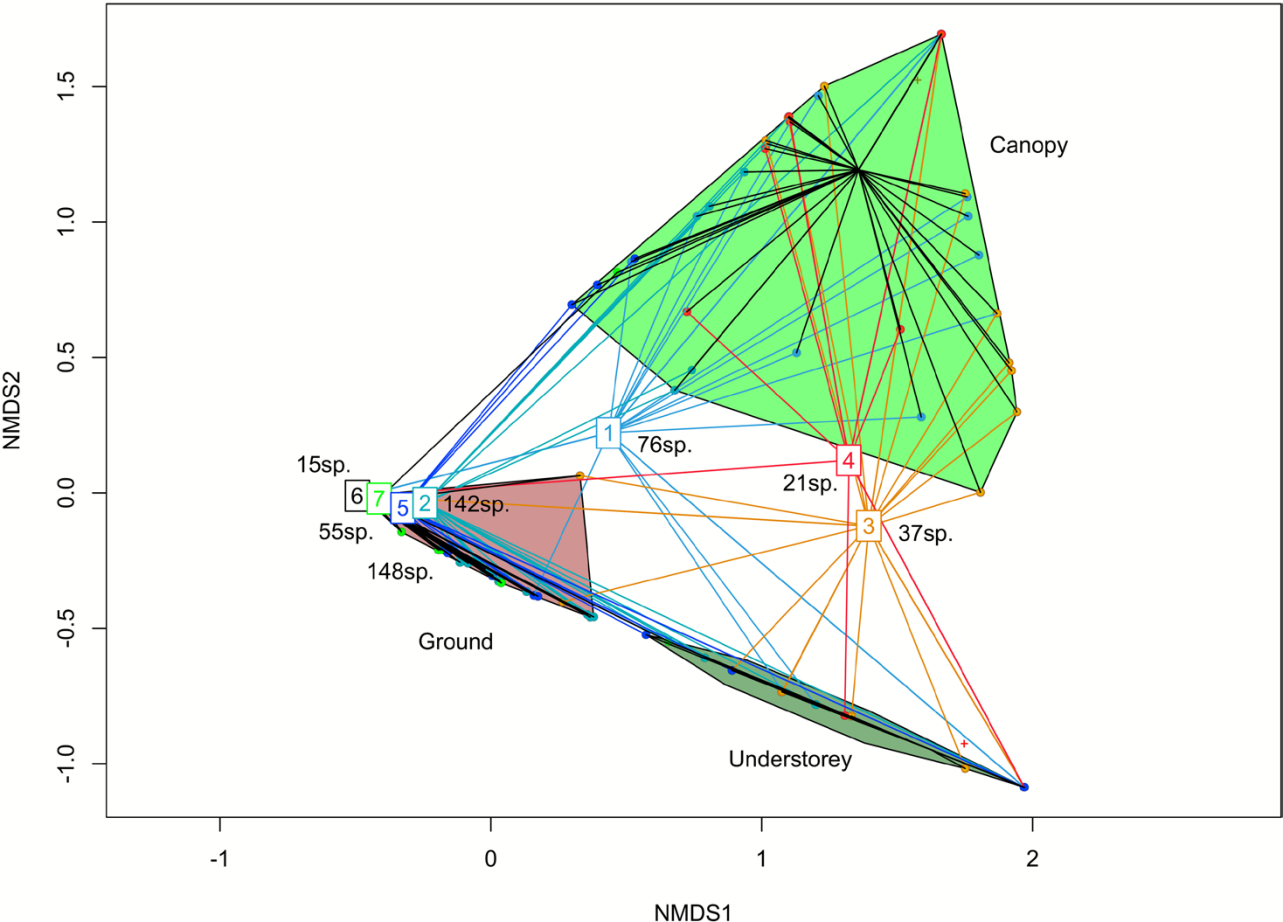
Nonmetric multidimensional scaling differentiating ground (brown), understorey (dark green) and canopy (green) species onto which we transposed the results of the Fuzzy Correspondence Analysis.

The NMDS analysis showed a clear separation between ant species from the ground, the understorey, and the canopy (Figure 6). The position of the functional trait clusters and their connections with those of the NMDS (i.e., forest strata) permitted us to note the following: Ants from cluster 1 (i.e., the hodgepodge) have connections with ants from the three forest strata. All ground‐dwelling ants are well grouped (i.e., clusters 2, 5, 6, and 7). The position of clusters 3 and 4 (understorey and canopy species, respectively) is relatively close to each other (Figure 6). Indeed, ants from cluster 3 have connections with ground‐dwelling ants mostly due to the leaf litter accumulated between the fronds of the palm trees, as well as with certain species from the canopy. Workers of different species of canopy ants are sometimes recorded on the ground. For the connections between ants belonging to the three forest strata, see Table S1.

## 4 Discussion

### 4.1 Ant Diversity

Thanks to their stratification, tropical rainforests are complex ecosystems where light availability and gradients of temperature and humidity allow numerous animal taxa to thrive, something particularly true for ants (Yanoviak and Kaspari 2000). Indeed, this study, conducted over approximately 3 ha using two techniques (i.e., Winkler extraction for ground‐dwelling ants and direct observations for understorey and canopy species, but requiring a climber in the latter case), enabled us to record 494 species, representing 75.1% of the 659 known Guianese ants (Franco et al. 2019) with an estimation of 566–670 species present. These results are comparable to the 489 species recorded in another Amazonian rainforest in Ecuador where subterranean, ground‐dwelling, and canopy ants were collected using six techniques; however, the estimation there was greater, ranging from 647 to 736 species (Ryder Wilkie et al. 2010). This similarity extends to the Mesoamerican forests of Panama as 405 species were noted in San Lorenzo, the estimation being 459–564 species (Leponce et al. 2021), and 539 species observed in the rainforest of La Selva Biological Station, the estimation reaching 584–639 species (Longino and Colwell 2020).

In Ecuador and Panama, fogging was employed to sample canopy ants, revealing substantial diversity in the canopy as this technique allows for the collection of various species that nest in rough trunk bark and/or are associated with epiphytes. However, the differences are significant enough that the sampling techniques alone cannot account for them: 56 species noted in the canopy in French Guiana (see Table S1) versus 282 species in Ecuador, and 182 and 261 species in Panama (Ryder Wilkie et al. 2010; Longino and Colwell 2020; Leponce et al. 2021). These differences are likely due to climatic perturbations that, although not very strong, are able to cause numerous trees in the Nouragues to fall (see Charles‐Dominique et al. 1998). In contrast, trees in other Neotropical rainforests tend to live longer, so that some of them bear numerous epiphytes (e.g., orchids and bromeliads), many of those sheltering ants (Rico‐Gray and Oliveira 2007). It is noteworthy that in the forested area of the Nouragues, epiphytes were limited to the ant gardens sheltering the TDAAs *Crematogaster levior* and *Camponotus femoratus*, which generally live in a parabiotic association, while other epiphytes were scarce (this study; Dejean et al. 2019).

### 4.2 Stratification and Ant Species Richness

The comminution of leaf debris by numerous detritivorous arthropods, by enhancing the activity of nutrient‐cycling microorganisms, aids in the decomposition of leaf litter fundamental to soil fertilization. These nutrients subsequently become available to the roots of trees that primarily develop in the topsoil layer. Consequently, the ground layer of tropical rainforests hosts large communities of detritivorous arthropods regulated by the top‐down effect of predators, particularly ants, which indirectly accelerates litter decomposition (Ashford et al. 2013; Basset et al. 2015; McGlynn and Poirson 2012). Moreover, species‐rich, omnivorous, ground‐dwelling ants can be abundant enough to move any material that can serve as food, including seeds, thereby redistributing nutrients and impacting decomposition and, consequently, the soil composition (Anjos et al. 2019; Griffiths et al. 2018).

Due to the height of Neotropical trees, which have comparatively modest trunk diameters, the permanent trade winds cause their crowns to touch, resulting in broken branches and the formation of gaps between trees. When TDAA colonies occupy several tree crowns that are not in contact, their workers descend in columns (trails followed by thousands of individuals) to the ground where they interconnect at the base of the trees, which explains their presence in Winkler extractors (see Table S1). Moreover, the number of ant species trapped there is further augmented by arboreal ants that descend from their host trees to forage in the litter (see Groc et al. 2017).

Thus, we noted 427 ground‐ and litter‐nesting ant species out of the 494 recorded in total, highlighting their impressive abundance, which is attributed to the occurrence of a range of arthropod prey taxa, something that contributes to strong ant diversification through various modes of predation. Additionally, these ants can be fungivores, omnivores, scavengers, generalists, or specialist predators (Figure 5a,b).

The understorey, with 98 ant species recorded, exhibited a much lower diversity than the ground but larger than that in the canopy (Figures 2 and 3). In this layer, numerous litter‐dwelling ants nest in the debris accumulated between the fronds of the palm tree *Astrocaryum* in a situation similar to Panama where a small palm tree also grows (Dejean et al. 2025; Leponce et al. 2021). Notably, colonies of the arboreal ponerine species *Od. hastatus* are recorded in 40% of the *Astrocaryum* that likely attract them, as do certain large epiphytes (Camargo and Oliveira 2012; Gibernau et al. 2007). Furthermore, all eight myrmecophytes noted in this forest stratum are associated with ants (Table S1). *Maieta* primarily shelters *Ph. minutula* whose queens are specifically attracted to it (Dáttilo et al. 2009; Vasconcelos 1993). *Hirtella* and *Cordia* mainly host *Allomerus decemarticulatus* and *A. octoarticulatus* , respectively. In both cases, *Allomerus* workers construct gallery‐shaped traps along the stems of their host plants to capture flying insects (Dejean et al. 2024). *Cecropia obtusa* houses several *Azteca* species, including *Az. alfari* and *Az. ovaticeps* that feed on the food bodies produced by the trichilia situated at the base of each petiole (Marting et al. 2018). *Tachigali paniculata* is generally associated with different *Pseudomyrmex* species, *Tococa* mostly with *Az. bequaerti*, and *Duroia* with different *Azteca* species (Dejean et al. 2025; Ward 1999).

Most myrmecophytes provide their associated ants with extrafloral nectar with production varying between day and night according to the plant's phenology (Juárez‐Juárez et al. 2023). *Cecropia* spp. supply their *Azteca* ants with food bodies (see above). In contrast, *Tachigali* spp. do not provide food to their associated *Pseudomyrmex* that feed on fungi developing in the domatia or on the honeydew produced by the mealybugs that they tend (Blatrix et al. 2012; Pacheco et al. 2022). *Tachia guianensis* is a nonspecialized myrmecophyte that also does not offer food to its associated ants, but instead obtains nutrients from them (Dejean et al. 2025). Indeed, plant ants provide nutrients to their host myrmecophytes (myrmecotrophy) via their refuse and feces (Gomes et al. 2021; Leroy et al. 2017; Orivel et al. 2017).

The canopy was mostly occupied by TDAAs, a phenomenon already known for this area (Dejean et al. 2019). Among them, we saw above that *Cr. levior* and *Ca. femoratus* were very frequent and typically associated in parabiosis; they build carton nests onto which they plant ant‐garden epiphytes (Vantaux et al. 2007). The presence of TDAAs in the rainforest canopy is facilitated by the abundance of the honeydew‐producing, sap‐sucking scale insects (Hemiptera) they tend (Davidson et al. 2003; Blüthgen and Stork 2007). Additionally, canopy lianas produce extrafloral nectar mostly exploited by nondominant ants forming the core of the species richness in this stratum (Adams et al. 2019; Blüthgen et al. 2000).

### 4.3 Ant Functional Traits and the Three Rainforest Strata

The grouping of the ants into seven clusters based on their functional traits follows a certain logic, although some differences arise from the interplay between their diet, nesting mode, and colony size (Leponce et al. 2021; Figure 5a,b; Table S2). Therefore, well‐defined feeding habits are grouped. This is true for all specialist predators, with small ground‐ or litter‐nesting colonies grouped in Cluster 7. In contrast, *Neoponera commutata* and *Ne. laevigata*, both of which have larger nests, belong to Cluster 5 despite being specialist predators of termites (Mill 1982, 1984). All 15 species showing an army ant‐like behavior are grouped in Cluster 6, which includes 14 Dorylinae plus the Ponerinae *Si. pergandei*, known for its phasic cycle and nomadic, army ant‐like behavior (Mackay and Mackay 2008). All the fungus‐growing ants are found in Cluster 5; however, ground‐dwelling ants, most of which have relatively small colonies, also belong to this cluster. All canopy ants (TDAAs) are grouped in Cluster 4 due to the large size of their populations; however, *Wa. rochai*, a ground nester with large colonies, also belongs to this cluster. Similarly, Cluster 3 includes all the understorey plant ants, along with *Od. hastatus* associated with palm trees, in addition to some arboreal ants characterized by the size of their colonies (1000–10,000 individuals); among them, *Neoponera villosa* is an arboreal Ponerinae common in the understorey of all Neotropical forests (Fernandes et al. 2014; Rocha et al. 2020). Cluster 2 encompasses ground‐ and litter‐nesting generalist feeders or scavengers and generalist predators with small colonies (300–1000 individuals), along with seven ponerine *Neoponera* able to live arboreally in hollow twigs, epiphyte domatia, or epiphytes (see Mackay and Mackay 2010), and *Pl. sinuata*, which nests in bromeliad epiphytes (DaRocha et al. 2015). Finally, Cluster 1 represents a sort of hodgepodge, as it groups generalist‐feeding ants whose colonies can be arboreal or ground‐ and litter‐dwelling, ranging from relatively small (300–1000 individuals) to very large (up to 100,000 individuals for *Azteca chartifex* , *Solenopsis saevissima* , and *Wasmannia auropunctata* ). Notably, *Pa. clavata* and *Ec. tuberculatum*, which nest in the ground at the bases of the trees where they forage, also belong to this cluster (Belk et al. 1989; Wheeler 1986).

The NMDS showed a clear differentiation of ant species between the ground, the understorey, and the canopy, permitting us to illustrate the position of the above‐cited functional traits in the forest strata.

In conclusion, within the broader context of tropical rainforests potentially losing their ant diversity due to climate change and other major threats (Almeida et al. 2025), this study instead shows that Neotropical rainforests are species‐rich, characterized by a complex network of specialized or generalized niches. Although we did not use insecticide fogging, it is likely that the structure of the forest in the region of our study limits the development of many epiphytes, resulting in the low number of associated ants observed. Indeed, a substantial portion of the ant species present establishes their nests on the ground or in the leaf litter, with some nesting in the litter trapped between the fronds of palm trees. Finally, a functional trait analysis further revealed seven distinct ecological clusters, highlighting the importance of diet for specialized species and their nesting habits, while colony size also influences species distribution between clusters. These patterns illustrate the structural and behavioral diversity shaping ant communities across forest strata and underscore the importance of considering functional traits in tropical biodiversity studies.

## Author Contributions

**Jacques H. C. Delabie:** conceptualization (equal), data curation (equal), formal analysis (equal), funding acquisition (equal), investigation (equal), validation (equal), writing – original draft (equal), writing – review and editing (equal). **Alain Dejean:** conceptualization (equal), data curation (equal), formal analysis (equal), investigation (equal), methodology (equal), supervision (equal), validation (equal), writing – original draft (equal), writing – review and editing (equal). **Maurice Leponce:** data curation (equal), investigation (equal), methodology (equal), resources (equal), writing – review and editing (equal). **John T. Longino:** funding acquisition (equal), investigation (equal), validation (equal), writing – review and editing (equal). **Jérôme Orivel:** funding acquisition (equal), investigation (equal), methodology (equal), validation (equal), writing – review and editing (equal). **Axel Touchard:** funding acquisition (equal), investigation (equal), validation (equal), writing – review and editing (equal). **Arthur Compin:** formal analysis (equal), methodology (equal), software (equal), writing – review and editing (equal).

## Funding

Financial support for this study was provided by research grants from the *Brazilian Conselho Nacional de Desenvolvimento Científico e Tecnológico* (CNPq‐306885/2023‐9) for J.H.C.D., the French *Agence Nationale de la Recherche* (CEBA, ref. ANR‐10‐ LABX‐25‐01), and the USA National Science Foundation grant DEB‐1932405 (Ants of the World) for J.T.L.

## Conflicts of Interest

The authors declare no conflicts of interest.

## Supporting information


**Table S1:** Ant subfamilies, tribes, and species recorded in the three rainforest strata of the Nouragues field station.

**Table S2:** Details of the different clusters based on functional traits and the rainforest strata.

## Data Availability

The data are inserted into the manuscript as Tables S1 and S2. They are accessible to the reviewers. They may be published as an appendix to the main document or made publicly available on a platform, as the publisher decides. Furthermore, the data that support the findings of this study are openly available in Figshare at https://doi.org/10.6084/m9.figshare.29071367.v1.
