# Supplementary material for: Ant Diversity and Stratification in an Amazonian Rainforest
Source: Ecol Evol. 2025 Dec 17;15(12):e72793. doi: 10.1002/ece3.72793 (PMC12710436; doi:10.1002/ece3.72793)
Supplement: Supplementary file 1 — Table S1: Ant subfamilies, tribes, and species recorded in the three rainforest strata of the Nouragues field station. [file ECE3-15-e72793-s002.docx]

Table S1 Ant subfamilies, tribes, and species recorded in the three rainforest strata of the Nouragues field station.

|  |  |  | **GROUND** | **UNDERSTORY** | | **CANOPY** |  |
| --- | --- | --- | --- | --- | --- | --- | --- |
| **Subfamily** | **Tribe** | **current classification** |  | ***Astrocarium*** | **Myrmecophytes** |  | **Total** |
| **Amblyoponinae** | Amblyoponini | *Fulakora lurilabes* | **8** | **0** | **0** | **0** | 8 |
| **Amblyoponinae** | Amblyoponini | *Prionopelta amabilis* | **4** | **0** | **0** | **0** | 4 |
| **Amblyoponinae** | Amblyoponini | *Prionopelta antillana* | **9** | **0** | **0** | **0** | 9 |
| **Amblyoponinae** | Amblyoponini | *Prionopelta dubia* | **4** | **0** | **0** | **0** | 4 |
| **Dolichoderinae** | Dolichoderini | *Dolichoderus abruptus* | 0 | 2 | **0** | 2 | 4 |
| **Dolichoderinae** | Dolichoderini | *Dolichoderus attelaboides* | 6 | 2 | **0** | 2 | 10 |
| **Dolichoderinae** | Dolichoderini | *Dolichoderus bidens* | 1 | 0 | **0** | 7 | 8 |
| **Dolichoderinae** | Dolichoderini | *Dolichoderus bispinosus* | 8 | 0 | **0** | 14 | 22 |
| **Dolichoderinae** | Dolichoderini | *Dolichoderus decollatus* | 0 | 0 | **0** | 7 | 7 |
| **Dolichoderinae** | Dolichoderini | *Dolichoderus diversus* | 0 | 0 | **0** | 2 | 2 |
| **Dolichoderinae** | Dolichoderini | *Dolichoderus gagates* | 0 | 0 | **0** | 5 | 5 |
| **Dolichoderinae** | Dolichoderini | *Dolichoderus imitator* | 11 | 2 | **0** | 0 | 13 |
| **Dolichoderinae** | Dolichoderini | *Dolichoderus laminatus* | 0 | 0 | **0** | 2 | 2 |
| **Dolichoderinae** | Dolichoderini | *Dolichoderus lutosus* | 2 | 0 | **0** | 2 | 4 |
| **Dolichoderinae** | Dolichoderini | *Dolichoderus* sp. cf*. luederwaldti* | 1 | 0 | **0** | 0 | 1 |
| **Dolichoderinae** | Leptomyrmecini | *Azteca alfari* | 0 | 0 | **5** | 0 | 5 |
| **Dolichoderinae** | Leptomyrmecini | *Azteca bequaerti* | 0 | 0 | **9** | 0 | 9 |
| **Dolichoderinae** | Leptomyrmecini | *Azteca brevis* | 0 | 0 | **4** | 2 | 6 |
| **Dolichoderinae** | Leptomyrmecini | *Azteca chartifex* | 0 | 0 | **0** | 3 | 3 |
| **Dolichoderinae** | Leptomyrmecini | *Azteca constructor* | 0 | 0 | **2** | 12 | 14 |
| **Dolichoderinae** | Leptomyrmecini | *Azteca depilis* | 0 | 0 | **7** | 0 | 7 |
| **Dolichoderinae** | Leptomyrmecini | *Azteca flavigaster* | 0 | 0 | **0** | 2 | 2 |
| **Dolichoderinae** | Leptomyrmecini | *Azteca instabilis* | 10 | 0 | **0** | 26 | 36 |
| **Dolichoderinae** | Leptomyrmecini | *Azteca jelskii* | 0 | 0 | **3** | 21 | 24 |
| **Dolichoderinae** | Leptomyrmecini | *Azteca muelleri* | 0 | 0 | **7** | 0 | 7 |
| **Dolichoderinae** | Leptomyrmecini | *Azteca ovaticeps* | 0 | 0 | **8** | 5 | 13 |
| **Dolichoderinae** | Leptomyrmecini | *Azteca schimperi* | 0 | 3 | **1** | 0 | 4 |
| **Dolichoderinae** | Leptomyrmecini | *Azteca* sp. nr. *xanthocrea* | 0 | 0 | **16** | 0 | 16 |
| **Dolichoderinae** | Leptomyrmecini | *Azteca* sp.01 gp. *chartifex* | 4 | 0 | **0** | 0 | 4 |
| **Dolichoderinae** | Leptomyrmecini | *Azteca* sp.01 nr. *constructor* | 0 | 0 | **2** | 0 | 2 |
| **Dolichoderinae** | Leptomyrmecini | *Azteca* sp.02 gp. *chartifex* | 2 | 0 | **2** | 0 | 4 |
| **Dolichoderinae** | Leptomyrmecini | *Azteca* sp.03 gp. *chartifex* | 3 | 0 | **13** | 0 | 16 |
| **Dolichoderinae** | Leptomyrmecini | *Azteca* sp.04 gp. *instabilis* | 0 | 0 | **3** | 1 | 4 |
| **Dolichoderinae** | Leptomyrmecini | *Azteca* sp.05 cf. *muelleri* | 0 | 0 | **2** | 0 | 2 |
| **Dolichoderinae** | Leptomyrmecini | *Azteca* sp.JTL-023 cf. angusticeps | 0 | 0 | **6** | 0 | 6 |
| **Dolichoderinae** | Leptomyrmecini | *Azteca sericeasur* | 0 | 1 | **0** | 0 | 1 |
| **Dolichoderinae** | Leptomyrmecini | *Dorymyrmex* sp. | 1 | 0 | **0** | 0 | 1 |
| **Dolichoderinae** | Leptomyrmecini | *Linepithema neotropicum* | 1 | 0 | **0** | 0 | 1 |
| **Dolichoderinae** | Tapinomini | *Tapinoma* sp.01 nr. *melanocephalum* | 1 | 0 | **0** | 0 | 1 |
| **Dolichoderinae** | Tapinomini | *Tapinoma* sp.02 | 1 | 0 | **0** | 0 | 1 |
| **Dolichoderinae** | Tapinomini | *Technomyrmex vitiensis* | 2 | 0 | **0** | 0 | 2 |
| **Dorylinae** | na | *Acanthostichus kirbyi* | 1 | 0 | **0** | 0 | 1 |
| **Dorylinae** | na | *Acanthostichus* sp. | 1 | 0 | **0** | 0 | 1 |
| **Dorylinae** | na | *Eciton burchellii* | 2 | 0 | **0** | 0 | 2 |
| **Dorylinae** | na | *Eciton drepanophorum* | 3 | 0 | **0** | 0 | 3 |
| **Dorylinae** | na | *Eciton hamatum* | 1 | 0 | **0** | 0 | 1 |
| **Dorylinae** | na | *Eciton vagans* | 1 | 0 | **0** | 0 | 1 |
| **Dorylinae** | na | *Labidus coecus* | 21 | 0 | **0** | 0 | 21 |
| **Dorylinae** | na | *Labidus praedator* | 3 | 0 | **0** | 0 | 3 |
| **Dorylinae** | na | *Neivamyrmex iridescens* | 1 | 0 | **0** | 0 | 1 |
| **Dorylinae** | na | *Neivamyrmex pilosus* | 1 | 0 | **0** | 0 | 1 |
| **Dorylinae** | na | *Neivamyrmex* sp.01 | 1 | 0 | **0** | 0 | 1 |
| **Dorylinae** | na | *Neivamyrmex* sp.02 | 1 | 0 | **0** | 0 | 1 |
| **Dorylinae** | na | *Neocerapachys splendens* | 1 | 0 | **0** | 0 | 1 |
| **Dorylinae** | na | *Nomamyrmex esenbecki* | 1 | 0 | **0** | 0 | 1 |
