# Supplementary material for: Ant Diversity and Stratification in an Amazonian Rainforest
Source: Ecol Evol. 2025 Dec 17;15(12):e72793. doi: 10.1002/ece3.72793 (PMC12710436; doi:10.1002/ece3.72793)
Supplement: Supplementary file 2 — Table S2: Details of the different clusters based on functional traits and the rainforest strata. [file ECE3-15-e72793-s001.docx]

Table S2 Details of the different clusters based on functional traits and the rainforest strata.

Arboreal ants are highlighted in light green. Some ponerine species in cluster 2 can be associated with hollow twigs or epiphytes (highlighted in a specific green). Plant-ants in Cluster 3 are highlighted in regular green, while *Odontomachus hastatus* associated with the palm tree *Astrocaryum sciophillum* is highlighted in dark green. In Cluster 4, *Wasmannia rochai* the lone ground-dwelling species of the cluster is highlighted in yellow to contrast with the other species that are arboreal. In Cluster 5, fungus-growing ants are highlighted in light yellow; *Neoponera commutata* and *N. laevigata* are highlighted in light orange as they are specialist predators, as are all species in Cluster 7.
